# Anaerobic Degradation of the Invasive Weed *Solidago canadensis* L. (*goldenrod*) and Copper Immobilization by a Community of Sulfate-Reducing and Methane-Producing Bacteria

**DOI:** 10.3390/plants12010198

**Published:** 2023-01-03

**Authors:** Olesia Havryliuk, Vira Hovorukha, Iryna Bida, Galyna Gladka, Artem Tymoshenko, Semen Kyrylov, Ruslan Mariychuk, Oleksandr Tashyrev

**Affiliations:** 1 Department of Extremophilic Microorganisms Biology, Zabolotny Institute of Microbiology and Virology of the National Academy of Sciences of Ukraine, 03143 Kyiv, Ukraine; 2 Department of Biotechnology, Faculty of Environmental Safety, Engineering and Technologies, National Aviation University, 03058 Kyiv, Ukraine; 3 Department of Ecology, Faculty of Humanities and Natural Sciences, Presov Universityin Presov, 08116 Presov, Slovakia

**Keywords:** *Solidago canadensis* L., goldenrod, carbohydrate content, anaerobic degradation of invasive weeds, copper, methane production, methane-producing bacteria, sulfate-reducing bacteria

## Abstract

The weed *Solidago canadensis* L. poses a global threat to the environment as it spreads uncontrollably on roadsides, in forests, fields, meadows, and farmland. Goldenrod emits toxic substances that suppress other plants on the site, displacing wild ones. Thus, goldenrod conquers huge areas very quickly. The use of herbicides and mechanical methods does not solve the problem of the spontaneous spread of goldenrod. On the other hand, many scientists consider goldenrod as a valuable source of biologically active substances: flavonoids, phenolic compounds, vitamins, etc. In this study, we consider *Solidago* plants as a promising, free (cheap), and renewable substrate for the production of methane gas. The goal of the study was to identify the main patterns of degradation of the *Solidago canadensis* L. plant by methane-producing and sulfate-reducing bacteria with methane gas production and simultaneous detoxification of toxic copper. The composition of the gas phase was monitored by gas chromatography. The pH and redox potential parameters were determined potentiometrically; metal concentrations were measured by photometry. The concentration of flavonoids, sugars and phenolic compounds in plant biomass was determined according to well-known protocols. As a result of the study, high efficiencies of methane degradation in the *Solidago* plant and copper detoxification were obtained. Methane yield has reached the value of 68.2 L kg^−1^ TS of *Solidago canadensis* L. biomass. The degradation coefficient (*K_d_*) was also high at 21.4. The Cu(II) was effectively immobilized by methanogens and sulfate reducers during the goldenrod degradation at the initial concentrations of 500 mg L^−1^. Thus, a new method of beneficial application of invasive plants was presented. The result confirms the possibility of using methanogenic microorganisms to produce methane gas from invasive weeds and detoxification of toxic metals.

## 1. Introduction

The *Solidago canadensis* L. weed poses a global threat to the environment as it spreads uncontrollably on roadsides, forests, fields, meadows and farmland [1]. Additionally, goldenrod releases toxic substances that suppress other plants (allelopathic effect), which leads to the displacement of wild ones [2]. Thus, goldenrod conquers huge areas very quickly. The use of herbicides and mechanical methods does not solve the problem of the spontaneous spread of goldenrod. For example, mowing and spreading fresh hay reduced *Solidago* cover. However, the *Solidago* cover was reduced only to 25% of the initial complete coverage during the six-year study period [3]. In addition, the plant biomass rots in landfills, which causes additional damage to the environment. In addition to harm, some scientists consider goldenrod as a valuable source of biologically active substances: green pesticides [4], flavonoids [5], phenolic compounds [6], vitamins [7], etc. An interesting aspect of the use of goldenrod is the production of biogas. The use of plant biomass to obtain energy carriers is already known. However, co-fermentation of plants with other organic substrates is usually carried out for the effective synthesis of biogas. The grass and cattle slurry co-fermentation was used to increase the effectiveness of biogas production [8]. This increases the costs of implementing the biotechnology and its operation. We consider the *Solidago canadensis* plants to be a promising, cheap, and renewable substrate for the production of methane gas without the use of co-substrate. This weed contains a huge concentration of organic substances, in particular carbohydrates [9], which will ensure high efficiency of biogas production. In this way, we solve two problems on a global level: the lack of energy carriers [10] and the utilization of ecologically hazardous plants [11,12]. We do not prefer the co-fermentation of different substrates [13,14] in this study. The *Solidago* plants contain a huge number of organic compounds (carbohydrates, cellulose, etc.) [15] that are sufficient for efficient anaerobic digestion. However, we consider co-cultivation to be a powerful tool for enhancing the diversity of microorganisms [16]. The use of an anaerobic microbial community to degrade plant biomass and produce methane is a logical and promising methodological approach [17]. Such microbial communities contain various groups of microorganisms (hydrolytic, acetogens, sulfate reducers, and methanogens) that are able to gradually transform organic plant polymers (cellulose, lignin, etc.) into the final products—methane and carbon dioxide [18]. A key role in biogas synthesis is played by methane-producing and sulfate-reducing bacteria, which coexist in a close syntrophic relationship, providing optimal conditions for existence [19]. However, hydrolytic and acetogenic bacteria create prerequisites for the growth of methanogens: they hydrolyze plant polymers and synthesize precursors for methane synthesis (organic acids, H_2_ and CO_2_) [18,20].

The *Solidago canadensis* plants are promising for biogas production for many reasons. They are widely distributed throughout the world and are able to spread across landscapes to reach favorable habitats and enter new communities (long-distance dispersal mechanisms) [21]. Thus, even without artificial sowing, these plants accumulate a large amount of biomass every year [22], which needs to be disposed of. At the same time, invasive plants can be considered bioenergy crops. The biomass of some invasive species has already been tested for its suitability for biogas production (biomethane [23] and biohydrogen [24]). The presence of a large number of organic compounds in goldenrod biomass, particularly carbohydrates, is a factor determining the efficiency of biogas production. The plant *Solidago canadensis* contains abundant biomass resources, such as high energy cellulose and lignin [15]. For example, Canadian goldenrod harvested in Slovenia contained 35% cellulose and 37% hemicellulose [25]. Cellulose is a carbohydrate consisting of β-D-glucose units, which can be consumed by microorganisms after decomposition into simple accessible sugars [26]. The process of *Solidago canadensis* biodegradation for methane production does not require the additional use of enzymes for hydrolysis, since anaerobic microbial communities are diversified and contain a large number of microorganisms of different physiological and taxonomic groups. These microorganisms have syntrophic chains [27] and are able to transform cellulose-containing substrates step by step with the involvement of various microbial species (acetogens, methanogens, sulfate reducers, etc.) [18,28].

Anaerobic degradation is a well-known bioprocess to convert a variety of biomass into value-added products, such as biohydrogen, biomethane, volatile fatty acids, etc. [29]. This process is extremely promising and economically beneficial for obtaining biogas [8]. However, its implementation is ensured by a diversified microbial community, which contains various physiological and taxonomic groups of microorganisms that gradually transform the available organic substrate into high-energy fuel. The conversion of organic biomass includes four main stages [18]:

1. Hydrolysis: [C_6_H_12_O_6_]_n_ (plant biomass) → C_6_H_12_O_6_ (*Bacillus*, *Bacteroides*, *Clostridia*)
2. Acidogenecis: 2 C_6_H_12_O_6_ = 3 CH_3_CH_2_COOH + 3 H_2_ + 3 CO_2_ (*Actinomyces, Bacillus*)
3. Acetogenesis: CH_3_CH_2_COOH + 2 H_2_O = CH_3_COOH + 3 H_2_ + CO_2_ + H_2_O
  (*Acetoanaerobacterium, Acetobacterium, Clostridium, Desulfotomaculum*)
4. Sulfate reduction [30]:
  2 CH_3_CHOHCOOH + SO_4_^2−^ = 2 CH_3_COOH + 2 HCO_3_^−^ + H_2_S
  4 H_2_ + SO_4_^2−^ + 2H^+^ = H_2_S + 4 H_2_O
  CH_3_COOH + SO_4_^2−^ = 2 HCO_3_^−^ + H_2_S (*Desulfovibrio* and *Desulfomicrobium*) [31].
5. Methanogenesis (*Methanosarcina* and *Methanosaeta*) [27,28]:
  5.1. Acetoclastic methanogenesis [32]: CH_3_COOH = CH_4_ + CO_2_
  5.2. Hydrogenotrophic methanogenesis [33]: 4 H_2_ + CO_2_ = CH_4_ +2 H_2_O.

Sulfate-reducing bacteria reduce sulfate with the formation of S^2−^. This ensures the existence of methanogens since they need reduced sulfur and do not carry out sulfate reduction (they lack the sulfate reductase enzyme). The symbiosis of SRB and MPB provides favorable conditions for their coexistence: the H_2_S consuming guarantees a suitable pH and S^2−^ concentration [34]. Herein, we also show the perspective of combining the processes of anaerobic degradation of plant biomass and metal detoxification.

Toxic metals pollution is another significant ecological problem that requires effective methods for its solution [35]. Metals are released into the environment at mining sites [36] and as a result of human industrial activity [37]. This causes the accumulation of metals in soils and groundwater and leads to inhibition of growth or even to the death of plants and microorganisms. Copper is a widespread contaminant that causes serious alterations in the metabolic functions of plants and microorganisms due to the excessive production of reactive oxygen [38]. Therefore, the development of methods for the simultaneous utilization of ecologically hazardous invasive plants, the synthesis of biogas, and the detoxification of metals is a promising scientific approach. These methods also require the isolation of promising metal-resistant microbial strains or microbial communities capable of copper detoxification [39].

The authors concluded, based on previous studies, that the biomass of invasive *Solidago canadensis* weed is a valuable energy source. Although the possibility of using *Solidago canadensis* for biogas production has already been studied, there is no data on the use of this weed as the main substrate (the only source of carbon and energy) for methane production. The use of anaerobic degradation of invasive plants for metal detoxification has not been fully investigated. Therefore, the aim of the work was to determine the main patterns of degradation of the *Solidago canadensis* L. invasive weed by methane-producing and sulfate reducing bacteria with methane gas production and simultaneous detoxification of toxic copper.

## 2. Materials and Methods

### 2.1. Sampling and Plant Biomass Preparation for Anaerobic Degradation

**Figure 1:**
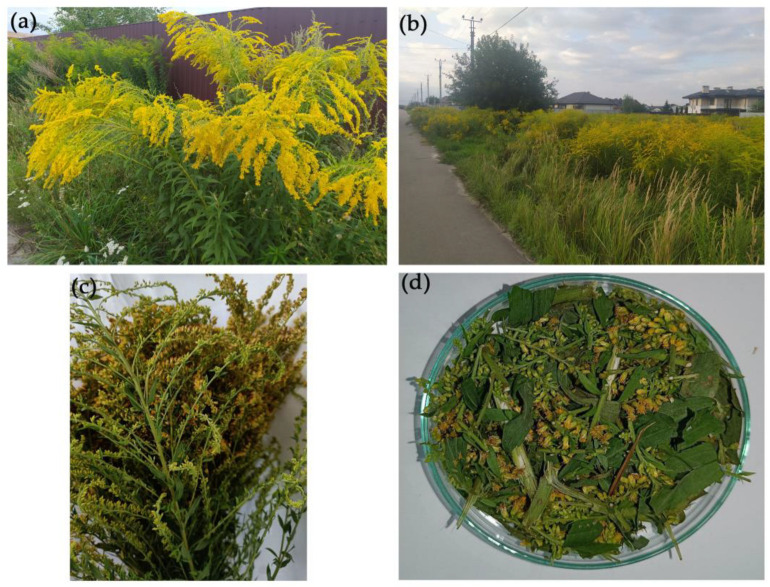
The spread of *Solidago canadensis* L. in the Sofiivska Borshchahivka village (**a**,**b**), Kyiv region, Ukraine) and drying (**c**) and the grinding (**d**) of plants for anaerobic degradation.

Canadian goldenrod (*Solidago canadensis* L.) plants were used as a substrate for methane production and metals detoxification. It is a perennial herbaceous honey-bearing plant of the family *Asteraceae* up to 1.5 m tall [40,41]. Biomass of goldenrod was sampled at Sofiivska Borshchahivka village (Kyiv region, Ukraine) in the phase of active flowering in August 2022 (Figure 1).

The plants were removed from the soil, cleaned, washed, and dried at room temperature to obtain a completely dry mass. To study the ability of methanogens to degrade goldenrod, it was ground to a size of 0.5–1 cm (Figure 1d).

### 2.2. Extraction of Solidago canadensis L. Bioactive Compounds

The plants were ground in a porcelain mortar to a powdery state before extraction. An extract was prepared in a 100 mL Soxhlet extractor using 10.2 g of air-dried plant material (whole plant) in 250 mL of 70% ethanol for 12 h within 50 cycles. The dry matter content of the obtained ethanol extracts was measured in five repetitions using the oven-drying method at 105 °C until constant weight [42].

### 2.3. Determination of Antioxidant Activity, Concentration of Phenols, Flavonoids and Total Carbohydrates in the Whole Plant of Solidago canadensis L. Weed

Antioxidant activity of extracts was determined via the DPPH-method with 2,2-diphenyl-1-picrylhydrazyl reagent [43]. The level of antioxidant activity was calculated in a percentage %. Total phenols were determined using the Folin-Ciocalteu reagent and expressed as mg of GA/g of extract in terms of gallic acid equivalent, GAE (the standard curve equation: y = 0.9727·x − 0.0037, r^2^ = 0.998) [44]. The amount of total flavonoids was determined by modified aluminum chloride colorimetric essay and expressed in rutin equivalent, RUE (the standard curve equation: y = 0.7791·x − 0.0115, r^2^ = 0.997) as mg of RUE/g of extract [45]. Determination of carbohydrates in the weed extract was carried out by the phenol-sulfuric acid method [44,46] (the standard curve equation: y = 1.5954·x − 0.0194, r^2^ = 0.992). The concentration of all biologically active substances was calculated per gram of the dry extract (mg per g of the extract).

### 2.4. Inoculum Preparation

Methane tank sludge was used as an inoculum for fermentation. It contained a large number of sulfate-reducing (SRB) and methane-producing bacteria (MPB) [47,48]. Samples of sludge were collected from a sewage treatment plant (Bortnychi Aeration Station, Kyiv region, Ukraine) in June 2022. Two variants of the inoculum were used for the experiment. The first is the native microbiome of the *Solidago canadensis* L. weed. The second was freshly sampled methane tank sludge containing a diversified community of SBR and MBR. The inoculum was added to the experimental jars in the amount of 5% of the medium at the beginning of fermentation. The presence of methanogens was confirmed experimentally by the appearance of CH_4_ [49] in the jars’ gas phase during degradation via the standard gas chromatography method [50]. The presence of sulfate-reducing bacteria was confirmed by the qualitative reaction of Fe^2+^ with the help of S^2−^ ions. Sulfate-reducing bacteria reduce sulfate (SO_4_^2−^) to sulfur reduced compounds, namely hydrogen sulfide (H_2_S) [51,52]. The presence of S^2−^ indicates the formation of black iron sulfide FeS↓ [53]. The cultivation was carried out in anaerobic conditions in hermetically sealed jars for 60 days. The initial pH and Eh values for the weed degradation were 7.2 and 6.9 as well as +238 mV and +212 mV in control and experimental conditions, respectively. The data were presented as mean values ± standard deviation (*n* = 3).

### 2.5. The Measurement of the Main Metabolic Parameters of Anaerobic Degradation of Solidago canadensis L. Weed

To study the effectiveness of the goldenrod degradation by a consortium of methane-producing and sulfate-reducing bacteria we used three variants of the experiment: The first was control variant (degradation of weed by native microbiome of *Solidago canadensis* without use of methanogenic and sulfate-reducing bacteria). The second variant was experimental (degradation of *Solidago canadensis* with microbial community dominated by methane-producing and sulfate-reducing bacteria). In the third variant, the microbial community of MPB and SRB was also used as an inoculum. However, copper was introduced in the anaerobic jar at the concentration of 500 mg L^−1^ in the active phase of degradation (28 days).

The following metabolic parameters were determined: concentration of the gas phase (CH_4_, CO_2_, %), cumulative gas production (mL), pH, Eh (mV), DOC (dissolved organic carbon, mg L^−1^). Before starting the experiment, the plant material was crushed. The degradation was carried out in anaerobic jars with volumes of 500 mL. The 20 g of absolutely dried and crushed *Solidago canadensis* weed and 400 mL of tap water were added to the jar. Degradation was carried out for two months (62 days) at 35 °C.

The H_2_, CH_4_ and CO_2_ concentrations were determined by the standard method on a gas chromatograph [54]. The redox potential (Eh) and pH of the medium were performed potentiometrically [55] using the EZODO MP-103 universal ionomer. The combined Ezodo ceramic chloride electrodes with BNC connectors–PY41 and PO50 models were used to measure pH and Eh. [56].

The permanganate method was used to determine the concentration of soluble organic compounds by the content of the dissolved organic carbon in the medium [57]. Carbon concentration was determined according to standard calibration curves: y = 0.11·x − 12.168, r^2^ = 0.994.

### 2.6. The Measurement of the Dynamic of Copper Detoxification during the Degradation of Solidago canadensis L. Weed

The ability of a consortium of MPB and SRB to detoxification of copper ions during goldenrod degradation was investigated.

The solution of 30,000 mg L^−1^ Cu(II) was used as the stock. It was prepared by CuSO_4_∙5H_2_O dissolving in distilled water in a volumetric flask. Citrate was used as a chelator to dissolve Cu(II). The Cu(II) concentration was determined by a colorimetric method with 4-(2-pyridylazo)resorcinol (PAR). The methods were based on the property of PAR to form colored red complexes with cations of bivalent heavy metals including Cu^2+^ [58].

Copper solution was added to the sealed glass jars on the 28th day of fermentation in concentrations of 100, 200 and 500 mg L^−1^ of Cu(II). The solutions were added sequentially, immediately after complete detoxification at the previous concentrations. The experiment was carried out in triplicate (*n* = 3). Standard deviation (SD) and average values (x) were calculated.

## 3. Results

### 3.1. Biochemical Composition of the Extracts of Solidago canadensis L.

Herein, we determined the concentration of certain biologically active compounds of plants that can affect the processes of their anaerobic degradation and copper detoxification [59]. Carbohydrates concentration is critical for efficient anaerobic degradation because they are the main source of carbon and energy for microbial growth and biogas synthesis [60]. Plants with high carbohydrate content are promising for biogas synthesis. Other important substances are phenolic compounds (flavonoids), in which the phenolic and carboxyl groups have reducing capacities and are able to reduce Cu^2+^ to Cu^+^ [61].

Whole-plant extracts using 70% ethanol were prepared to examine the antioxidant activity and concentrations of carbohydrates, phenols, and flavonoids. We observed that weed extracts contained a large amount of carbohydrates. The total concentration of carbohydrates in the goldenrod was 4.54 mg mL^−1^ of extract or 511.5 mg g^−1^ of dry matter. The concentration of phenolic compounds was also high and amounted to 4.3 mg of GAE mL^−1^ or 485.6 mg of GAE g^−1^ of extract. The amount of flavonoids was 3.4 mg mL^−1^ or 385.7 mg g^−1^ of extract.

The concentrations of bioactive compounds in the samples of goldenrod are presented in Table 1 and calculated per volume of extract (mg mL^−1^), as well as per dry weight of plants (mg g^−1^ of plant) and dry weight of extract (mg g^−1^ of extract).

The extract antioxidant activity was also high, reaching the value of 86.3 %. Thus, it is demonstrated that goldenrod plants contain a large concentration of organic compounds, in particular carbohydrates. We assumed that goldenrod biomass should be intensively fermented for the formation of biogas without the use of additional co-substrates.

### 3.2. Detoxification of Copper via Methane Fermentation of Solidago canadensis L. Weed Biomass

**Figure 2:**
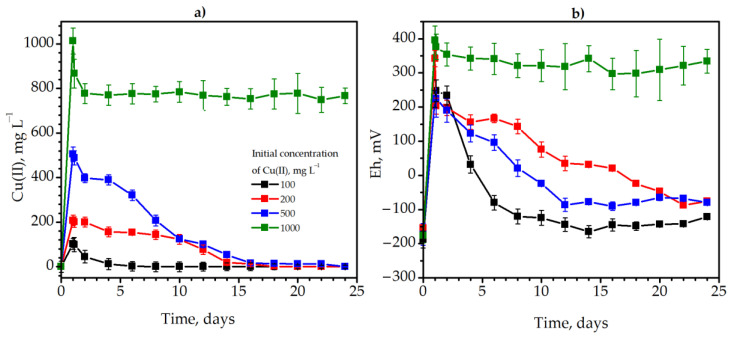
The dynamics of the Cu(II) detoxification (**a**) and Eh (**b**) during methane degradation of *Solidago canadensis* L. weed at the presence of 100 (black lines), 200 (red lines), 500 (blue lines), and 1000 (green lines) mg L^−1^ Cu(II): The black lines represent the value of the redox potential.

Copper was inserted into the anaerobic jar with tap water and *Solidago* plants in the active phase of microbial growth at the 28th hour of cultivation in concentrations of 100, 200, 500 and 1000 mg L^−1^. This was necessary to determine the concentration at which immobilization will still occur. Detoxification took place in the cultivation medium due to the metabolic activity of sulfate-reducing and methane-producing bacteria (inoculum). Methanogenic bacteria most likely reduced Cu(II) compounds to insoluble Cu_2_O, and sulfate-reducing microorganisms precipitated copper in the form of insoluble sulfides. The detoxification rate of copper and its efficiency at 100, 200, and 500 mg L^−1^ Cu(II) were extremely high. The fastest immobilization of copper occurred at the initial concentration of 100 mg L^−1^ Cu(II). It decreased from 102.4 to 2.1 mg L^−1^ on the sixth day of methane fermentation of goldenrod (Figure 2a, black lines). In total, immobilization lasted eight days with 100% effectiveness. At the initial concentration of 200 mg L^−1^ Cu(II), the duration of complete immobilization was higher and amounted to 18 days (Figure 2a, red lines). The concentration of Cu(II) decreased from 505.0 to 12.1 mg L^−1^ on the 22nd day after copper addition and was equal to 0 only on the 24th day (Figure 2a, blue lines).

The duration of detoxification was 24 days with 100% efficiency. In contrast to the previous variants, increasing the concentration of Cu(II) to 1000 mg L^−1^ had a detrimental effect on the anaerobic microbial community (Figure 2a, green lines). In two days, the concentration of soluble Cu(II) decreased only from 1013.5 to 777.5 mg L^−1^ (Figure 2a, green lines). Later, the copper concentration did not change, and the immobilization efficiency was only 23.3%.

The addition of copper significantly affected the redox potential of the media (Figure 2b). For example, the Eh increased sharply from −173 to 223 mV after the insertion of 500 mg L^−1^ Cu(II) (Figure 2b, blue lines). However, the Eh gradually returned to more optimal for anaerobic bacteria negative values after detoxification of copper in the medium. Similar patterns of redox potential dynamics were also observed at lower concentrations of copper (100 and 200 mg L^−1^). However, the Eh increased sharply to exceedingly high values +396 mV after copper insertion at the concentration of 1000 mg L^−1^ (Figure 2b, green lines) and remained at an elevated level (suboptimal for the growth of anaerobes).

Thus, it was determined that 500 mg L^−1^ of Cu(II) is a high concentration at which 100% immobilization of copper occurs. Therefore, it was of interest to investigate the influence of copper in such a concentration on the metabolic parameters of degradation of *Solidago canadensis* L. weed biomass by methane-producing and sulfate-reducing microbial community.

### 3.3. Anaeribic Degradation of Goldenrod by Methane-Producing and Sulfate-Reducing Bacteria

**Figure 3:**
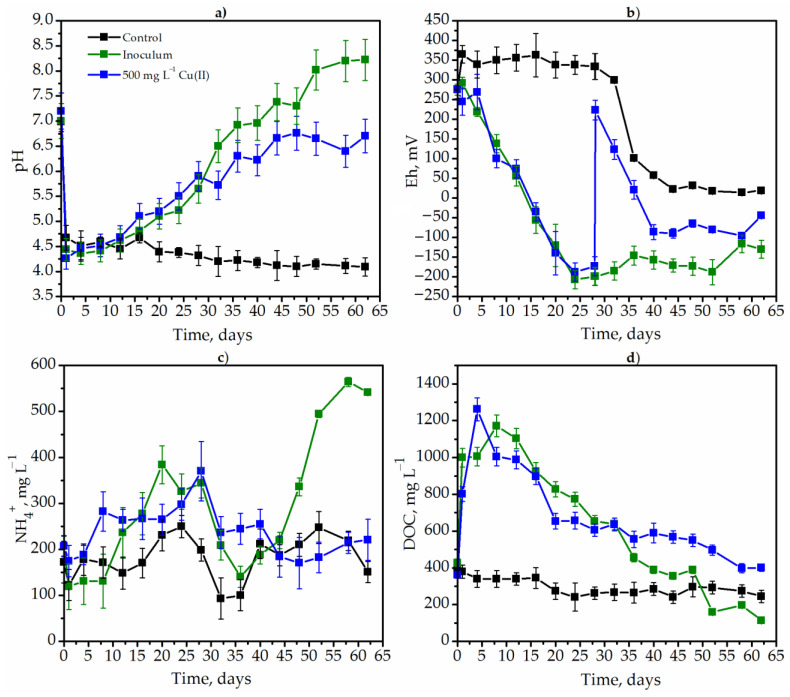
The dynamics of *Solidago canadensis* L. metabolic parameters pH (**a**), Eh (**b**), NH_4_^−^ (**c**) and DOC (**d**) in different experimental conditions: the degradation by native microbiome (control without metals and additional inoculum) (black lines); the degradation by methane-producing and sulfate-reducing bacteria (green lines); the degradation at the presence of 500 mg L^−1^ Cu(II) (blue lines).

The dynamics of pH, Eh dissolved organic carbon (DOC) and ammonium ions (NH_4_^+^) concentration were studied during the degradation of goldenrod weed both in control conditions and in the presence of a native microbiome (control), with additional inoculum (source of methane-producing and sulfate-reducing bacteria), as well as in the presence of toxic metals. The influence of metals on the metabolic parameters of weed degradation is presented using the example of copper (Figure 3).

The pH decreased sharply as a result of the synthesis of acidic exometabolites by the native microbiome of goldenrod under control variant of the experiment (Figure 3a, black lines). Thus, the pH value decreased from 6.99 to 4.7 in just one day. Subsequently, the pH also slowly decreased and was 4.1 on the 62nd day (Figure 3a). The microbial community of MPB and SRB adapted the pH to optimal values. In this case, the pH also decreased from 7.0 to 4.4 for four days. After that, it started to grow and was 6.5 and 8.02 on day 32 and day 52, respectively. The pH increased very slowly from 4.7 (12 h of degradation) to 6.6 (52 h of degradation) after copper insertion (the 28th hour of degradation).

The addition of copper significantly affected the redox potential (Figure 3b). The Eh increased sharply from −173 to 223 after the insertion of copper (Figure 2b). However, after copper detoxification in the medium, the Eh gradually returned to more optimal negative values for anaerobic bacteria. The redox potential did not significantly decrease to negative values under the degradation of goldenrod by the native microbiome (control variant). This indicates the low metabolic activity of microorganisms. The experimental variant with inoculum showed the most intense growth of obligate anaerobes. The Eh decreased from +275 to −120 mV on the 24th day of fermentation and remained at a low level until the end of degradation (Figure 3b).

The high metabolic activity of MPB and CRB was confirmed by the dynamics of DOC. The hydrolysis of plant polymers took place during the first few days, which was evidenced by an increase in the DOC concentration from 427.8 to 999.8 mg L^−1^ after one day of degradation. In contrast to this variant, no changes in DOC were observed in the control. The DOC consumption slowed down after the insertion of copper (Figure 3d). Ammonium ions accumulated very intensively in the variant with methanogens and sulfate reducers, starting from the 40th day of fermentation. Thus, the concentration of ammonium ions increased from 188.0 to 564.3 mg L^−1^. The concentration of ammonium ions remained approximately at a constant level in the control and under the influence of copper (Figure 3c). Thus, in the control conditions, the reason for inefficient fermentation was the lack of a specific inoculum substrate. In the variant with copper, the metabolic parameters stabilized immediately after detoxification in the solution.

### 3.4. Biogas Synthesis and the Efficiency of the Goldenrod Fermentation Process

**Figure 4:**
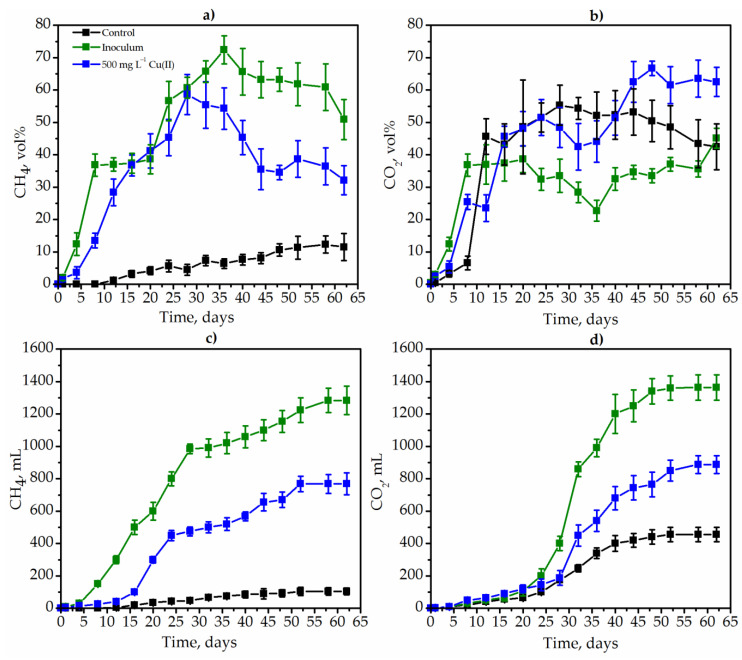
The dynamics of the concentrations of CH_4_ (**a**) and CO_2_ (**b**) during the degradation of *Solidago canadensis* L. weed: the degradation in control conditions with native microbiome (black lines), the degradation by inoculum (MPB and SRB, green lines) as well as under the influence of 500 mg L^−1^ Cu(II) (blue lines).

The main criteria for the evaluation of the efficiency of the anaerobic degradation of goldenrod were the concentration of methane in the gas phase, the volume of gas synthesized by microorganisms, as well as the decrease of the weed weight (*K_d_*). Methane (CH_4_) and (CO_2_) and carbon dioxide were the main gas phase during degradation of weed. Methane synthesis took place from the 12th day of degradation and amounted only to 1.2 vol% under control conditions (Figure 4a).

For 58 days of *Solidago* degradation, the maximum concentration of CH_4_ was 12.3 vol%. In contrast to the control conditions, in the version with the inoculum, the concentration of methane was extremely high and even reached 72.4 vol% on the 36th day of degradation. The concentration of CH_4_ decreased significantly after the addition of Cu(II) (Figure 4a, blue lines), but remained higher than in metal-free control conditions (Figure 3a, black lines). Thus, the maximum concentration of CH_4_ in the variant with copper was 58.6 vol% (Figure 4a, blue lines).

The CO_2_ concentration also increased during the whole degradation process. The least amount of carbon dioxide was synthesized in the version with the inoculum with a maximum concentration of 45.1 vol% on the 62nd day of degradation. CO_2_ concentration reached 66.7 vol% in the variant with copper (Figure 4b).

The effective degradation of weeds was evidenced by the high coefficient of degradation (K_d_) of *Solidago canadensis* L. weed. Thus, the variant with MPB and SRP inoculum had the highest degradation coefficient, reaching 21.4. Under control conditions, degradation almost did not occur (Table 2).

**Figure 5:**
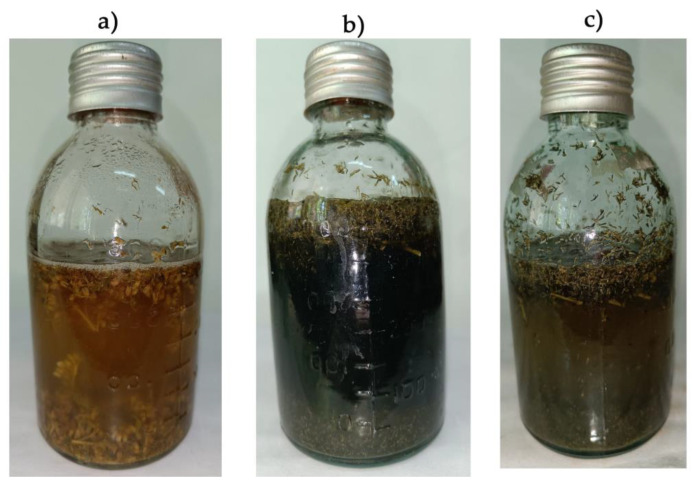
The process of *Solidago canadensis* invasive weed degradation (34 days) using different experimental treatments: control degradation by native *Solidago* microbiome (**a**); experimental degradation by inoculum of methane-producing and sulfate-reducing bacteria (**b**); degradation under the influence of 500 mg L^−1^ Cu(II) (**c**).

The process of *Solidago canadensis* weed degradation is presented in Figure 5.

Thus, herein is shown the effective degradation of goldenrod by methane-producing and sulfate-reducing bacteria with a high yield of methane. Methane-producing bacteria play a key role in degradation. In control conditions without additional substrate-specific inoculum, degradation did not occur, and biogas was not synthesized in significant quantities. Copper inhibited the fermentation process, but despite an extremely high copper concentration of 500 mg L^−1^, anaerobic microorganisms adapted and continued to grow and synthesize methane after complete metal immobilization.

## 4. Discussion

Biogas production via the fermentation of invasive environmentally hazardous plants is one of the promising fields considered in a number of studies [23,62]. The reasons for this are the significant energy potential of such plants and the high content of biologically active compounds, in particular carbohydrates [63]. In addition to carbohydrates, the invasive plants contain significant amounts of flavonoids, vitamins, and other antioxidants [64]. Therefore, the biomass of such plants was used as a valuable substrate for growth of anaerobic microorganisms and biogas synthesis. Invasive plants were used to produce biofertilizer [65], ethanol [66], and biofuels [67]. However, the direction of using such plants as a substrate for anaerobic degradation (fermentation) is almost non-existent.

The weed *Solidago canadensis* L. is extremely promising for obtaining biogas. It contains a huge amount of biologically active compounds as well as lignocellulosic biomass, [68] and has great potential for conversion into bioenergy [29]. The anaerobic co-digestion of *S. canadensis* via sewage sludge with different volatile solid ratios in feedstock was investigated [29]. However, this process is technologically complicated because there are additional co-substrates. Another example is the optimization of anaerobic co-digestion of *Solidago canadensis* L. biomass and cattle slurry [69]. In this report, the highest biomethane yield was 143.7 L kg^−1^ volatile solids [69]. In this case, the high yield of biomethane may have been caused by the presence of additional substrates for methanogens in the sewage sludge. In our case, the community of methane-producing and sulfate-reducing bacteria was used as an inoculum, and the biomass of *S. canadensis* was used as substrate (only one source of carbon and energy). In our experiment, without any additional substrates, the yield of biomethane was high (64.2 L kg^−1^ TS). In the available literature, there are no results on the anaerobic degradation of goldenrod without introducing additional components into the process. For example, in Latvia, “Metaferm” biocatalyst was used to increase the efficiency of fermentation of goldenrod and nettle [70]. In this case, it would be more appropriate to postpone co-fermentation and focus on co-cultivation of bacteria or the use of microbial communities to degrade the biomass of invasive plants or other wastes. Methanogenic microbial communities are highly promising for this purpose. Syntrophic metabolism in methanogenic microbial communities plays a crucial role in organic biomass conversion to biogas [19]. An ecologically significant group involved in syntrophic degradation of polymers are bacteria of *Desulfovibrio* genus. They degrade lactate, producing acetate and hydrogen in syntrophic associations with methanogens [19].

Herein, it was shown that SRB and MPB of fermented methane tank sludge efficiently fermented goldenrod biomass, while in control conditions without methanogens and sulfate reducers, fermentation almost did not take place.

Another environmental problem that needs to be solved is the pollution of the environment with heavy metals, in particular copper [19]. For the first time, we developed a methodology to solve three environmental problems at once—utilization of invasive plants, biogas synthesis, and metals detoxification. The investigated methanogenic microbial community also adapted to copper after its introduction in a high concentration (500 mg L^−1^). In the experimental conditions without copper, the yield of methane was 64.2 L kg^−1^ TS, and under the copper influence, it was 1.7 times lower 38.4 L kg^−1^ TS. However, this is a significant yield of methane given such extreme (copper stress) conditions for sensitive strict anaerobic bacteria.

Sulfate-reducing microorganisms are equally important for metal detoxification. The use of anaerobic microorganisms for metal detoxification is promising but little researched. However, some sulfate reducers that precipitate copper in the form of insoluble compounds (CuS↓) have already been investigated. Sulfate-reducing microorganisms reduce SO_4_^2−^ ions in the process of dissimilatory sulfate reduction and emit hydrogen sulfide H_2_S, which interacts with Cu^2+^ and precipitates it in the form of the insoluble sulfide CuS [71]. Microorganisms of the genus *Desulfuromonas* are able to immobilize Cu(II) in the form of CuS↓ using H_2_S with high efficiency that reached 97.3–100.0% [72]. According to well-known studies, heavy metals are toxic to methanogens [73]. However, they can be more stable and immobilize metals during growth in a microbial community [73]. We experimentally proved that the microbial community of methane-producing and sulfate-reducing bacteria is able to completely remove toxic copper compounds from solutions at high concentrations of Cu(II). It was theoretically substantiated that one of the most important mechanisms was the precipitation of copper sulfide due to the sulfate reduction associated with methanogenesis. Despite the unquestionable evidence of the high efficiency of the metal detoxification during methane fermentation by precipitation in the form of sulfides, these processes still remain poorly studied and require further investigations.

## 5. Conclusions

The obtained results showed the general opportunity to effectively use a consortium of methane-producing and sulfate-reducing bacteria for the methane degradation of environmentally hazardous invasive *Solidago canadensis* L. plants. Anaerobic methanogenic microorganisms were shown to be promising for the high-efficiency degradation of goldenrod and the synthesis of high energy carrier methane, as well as for the complete detoxification of toxic Cu^2+^ from solutions where it is high in concentration. Sulfate-reducing and methane-producing bacteria analysis, economic calculations, and industrial modeling will be the next important steps to develop the optimal method of simultaneous hazardous invasive weeds degradation, copper detoxification, and obtaining of biofuel.

## Figures and Tables

**Table 1:** Total phenol, flavonoids and carbohydrates contents in the plant extracts and plant biomass of *Solidago canadensis* L.

Type of Analysis	Value mg mL^−1^ of Extract	Value mg g^−1^ of Extract	Value mg g^−1^ of Plant
Phenols (GAE)	4.3 ± 0.3	485.6 ± 28.4	105.7 ± 6.2
Flavonoids (RUE)	3.4 ± 0.1	385.7 ± 16.4	84.0 ± 3.6
Total carbohydrates	4.5 ± 0.2	511.5 ± 23.1	111.4 ± 5.0
DOC	8.5 ± 0.5	956.8 ± 45.5	208.33 ± 17.7
Antioxidant activity, %	86.3 ± 4.2	-	-

**Table 2:** The effectiveness of the fermentation process in the presence of Cu(II).

Treatments	CH_4_ Max (vol%) *	CH_4_ Yield (L kg^−1^ TS_plant)_	CO_2_ Yield (L kg^−1^ TS_plant)_	*K_d_* (Times)
Control with native bacteria	12.3 ± 2.6	5.2 ± 1.3	22.8 ± 3.5	1.5 ± 0.7
Inoculum of MPB ^#^ and SRB **	72.4 ± 4.3	64.2 ± 9.1	68.2 ± 12.2	21.4 ± 3.2
500 mg L^−1^ Cu(II)	58.6 ± 6.2	38.4 ± 4.6	44.4 ± 11.5	7.4 ± 5.1

Within each row, the means (±SD, *n* = 3). * Maximum CH_4_ concentration. *#* Methane-producing bacteria. ** Sulfate-reducing bacteria.

## Data Availability

Not applicable.
